# Building urban resilience through sustainability-oriented small- and medium-sized enterprises

**DOI:** 10.1186/s42854-022-00041-9

**Published:** 2022-07-28

**Authors:** Sarah Burch, Jose DiBella, Arnim Wiek, Stefan Schaltegger, Wendy Stubbs, Megan Farrelly, Barry Ness, Kes McCormick

**Affiliations:** 1 University of Waterloo, Waterloo, Canada; 2 Arizona State University, Tempe, USA; 3 Leuphana University, Lüneburg, Germany; 4 Monash University, Melbourne, Australia; 5 Lund University, Lund, Sweden

## Abstract

The unfolding COVID-19 pandemic, and the unprecedented social and economic costs it has inflicted, provide an important opportunity to scrutinize the interplay between the resilience of small and medium-sized enterprises (SMEs) and the resilience of the communities they are embedded in. In this article, we articulate the specific ways that SMEs play a crucial, and underappreciated role in building resilience to human and natural hazards, and provide new opportunities to accelerate the adoption of sustainability practices through the configuration of ‘enabling ecosystems’ geared towards promoting sustainability in the private sector. We argue that capacity-building and experimentation are not only required within companies, but also throughout this emerging supportive ecosystem of policies, resources (i.e. finance, materials, skills), governance actors, and intermediaries to adequately focus investment, technical capabilities and innovation. Ultimately, we call for a new transdisciplinary action research agenda that centers on SMEs as pivotal actors and amplifiers of community resilience; while recognizing that these firms are themselves in need of support to secure their own capacity to respond to, and transform in light of, crises. This research program calls for recognizing and applying the lessons that the pandemic presents to the urgent need for accelerated climate action. This will be enabled by developing more targeted approaches to collaborative capacity-building activities in SMEs that feed into experimentation and allow for the accelerated adoption of deliberate and strategic resilient business practices and models.

## Policy and practice recommendations

- An emerging agenda for policy and practice should center on SMEs as pivotal actors and amplifiers for community resilience
- Capacity must be built within the supportive ‘ecosystem’ of policies, resources (i.e. finance) governance actors, and intermediaries, not simply within SMEs
- To enhance the transformative potential of SMEs, support programs and policies should target the underlying business model rather than only on resource efficiency

## Sustainable SMEs’ contributions to building resilient pathways

The impact of the COVID-19 pandemic on small and medium-sized enterprises (SMEs)^1^ parallels that of a large-scale environmental disaster. Initial pandemic restrictions took a heavy toll on small firms, but even after the mandated closures eased, businesses in many regions faced a shortage of personal protective equipment, the inability of employees to return to work, disrupted supply chains, reduced market demand (from the global to local scales), cancellation of orders and absence of new orders, significant cash flow issues due to fixed expenditures and little or no revenue. As a result, SMEs had difficulties in repaying loans and interest (Lu et al. 2020; Robinson and Kengatharan 2020) placing unprecedented pressures on operations of many SMEs. These impacts are not uniform across the vast diversity of small firms, however, with construction, hospitality and the arts facing some of the most serious declines (Belitski et al. 2022; Fairlie 2020).

Similar challenges occur in the context of disasters and natural hazards, which are becoming more frequent and severe in a changing climate. SMEs are disproportionately affected by environmental risks compared to larger firms due to financial constraints, limited management and human resources, focus on short-term planning and survival with a hierarchical management culture, and lack of time and/or skills to prepare for environmental challenges (Halkos and Skouloudis 2020). While small enterprises usually do not have the financial capacities to invest in research on preparing for and mitigating environmental risks (Ryu et al. 2017), especially those that may intensify or shift course in the future, medium-sized organizations may have more financial and technical resources. There is value, however, in examining this varied and diverse group: they are fundamentally different than their larger corporate counterparts, they have been slower to develop and implement ambitious sustainability interventions, and they are crucial drivers of economic prosperity (DiBella et al. 2022; Westman et al. 2021). These observations raise several questions: what is the nature of the relationship between the structure and function of SMEs, their ability to withstand or even thrive in the face of shocks, and the resilience of the community of which they are a part? How might that resilience connect to the broader transformation towards sustainable development pathways (Westley et al. 2011)?

## Exploring the links between SME and community resilience

For SMEs to build resilience to human and natural hazards and prevent disasters, capacity must be built not only within the companies and their organizational structures and processes, but also within the supportive ‘ecosystem’ of policies, resources (i.e. finance), governance actors, and intermediaries. SMEs act as pivotal amplifiers that can enhance resilience in the communities they serve and are embedded in. While many definitions of community exist, we think of community as the dynamic interplay among the place-based physical and natural environment, and the constellation of civil society organizations, individuals, government actors, and broader private sector entities within which a business operates (see for example Howard et al. 2022). This may differ from a strict political jurisdiction (the boundaries of a municipality for instance), and encompasses more than a simple supply chain or set of customers. More recent threads in resilience thinking elaborate upon this interdependency of elements of a system (Scoones et al. 2020) and the ways that transformation may unfold either as carefully planned or more unpredictable, emergent processes (cf. Stirling 2015).

Likewise, the resilience of SMEs emerges out of, and is deeply rooted in, broader community resilience (Steiner and Atterton 2014). This bidirectional relationship of mutual interdependency and support suggests that it is unhelpful to view SMEs as rational, economic ‘islands’ untouched by the rich socio-political context within which they exist, but rather an integral part of it. Indeed, resilience (the capacity of a system to withstand or even adapt and innovate in response to shocks while maintaining its function and purpose) (Holling 1973; Folke 2006) is one quality of a sustainable society (a normative proposition describing a just, prosperous, socio-ecological state that respects the rights of both future generations and non-human nature) (cf. Elmqvist et al. 2019). Sustainable SMEs need also to be resilient, dynamically responding to crisis and opportunity, while playing a role in the broader community of which they are a part.

Small businesses are deeply embedded in their host communities and play a crucial role in the local economy. SMEs often serve as fixtures in the community, contributing to local well-being, reducing vulnerability to disasters, and facilitating disaster recovery of local communities (Halkos et al. 2018; Linnenluecke 2017; Price et al. 2013). SMEs can diversify the local economy, help to build wider community resilience by delivering a range of products and services that are essential for the well-being of communities and create jobs for community members (Steiner and Atterton 2014).

As innovation incubators and learning hubs, SMEs can “respond flexibly and adapt to higher levels of disorder and change” (Halkos and Skouloudis 2020, p. 58) than their larger corporate counterparts. SMEs are incredibly diverse, however, and the size, make-up and sector of the firm deeply influences the sustainable practices at play (Bakos et al. 2020). Extremely small or new firms face greater challenges related to technical and financial capacity, while larger or more established firms encounter organizational complexity and inertia (but more significant resources) (Balasubramanian et al. 2021). Sustainability interventions also vary widely by sector: manufacturing or construction firms may prioritize more efficient resource use (for instance with ‘lean’ thinking and strategies – cf. Caldera et al. 2019), supply chain sustainability, and mitigation of waste, while companies in the arts, professional services, or educational sectors may focus on employee engagement, awareness, and community-building. SMEs using a cooperative business model (e.g., employee-owned and customer-owned), have proven particularly resilient in times of crisis, maintaining the livelihoods of the communities in which they operate (Birchall and Ketilson 2009). However, SMEs do not automatically enhance community resilience simply through their existence. Dedicated efforts to build partnerships and capacities are necessary for SMEs to be able to fulfill this function.

Partnerships and collaboration are essential for SMEs to be able to enhance community resilience during times of crisis. In ‘normal’ times, multi-stakeholder partnerships and information or expertise-sharing with local stakeholders (e.g., local government, civil society organizations) enable SMEs to access partners’ innovation resources and overcome SMEs’ intrinsic resource constraints, while developing resilience-specific innovations within and beyond the SMEs (Ahn et al. 2018; Alcalde-Heras et al. 2019; Halkos et al. 2018). During times of crisis, SMEs can then utilize their partners’ social networks (social capital) (Aldrich and Meyer 2015; Fraser et al. 2021) and access important resources for communication, coordination, and support (McNaughton and Gray 2017). Internal collaboration and empowerment of the workforce (e.g., in worker cooperatives) enable SMEs to self-organize in times of crisis, which in return allows them to assist the community in meeting their recovery needs (Thomas et al. 2012).

Adaptability and flexibility are also significant capacities to be built for fostering the resilience of SMEs and, in turn, the community (Andres and Round 2015; McNaughton and Gray 2017). Such capabilities allow SMEs to successfully operate even if there are significant fluctuations in supply or demand as well as to respond to new requirements through changes to business practices and models. More especially in SMEs that have adopted sustainability as core to their business model, several types of practices have been identified to provide unique opportunities to connect with resilience building process at local scales, such as strategic placemaking, integration of novel and varied skillsets in their organizational structures and a stronger focus on employee empowerment and ownership (Burch and Di Bella 2021; Burch et al. 2020). Such SMEs might be viewed as ‘sustainability-oriented’ when they place the ecological and social good on par with economic profit, with societally beneficial outcomes woven throughout both their purpose and activities.

Again, these traits allow SMEs to not only survive in times of crisis but also lend a hand to community organizations and groups as needed. SMEs can also strengthen their own resilience as well as their communities’ resilience through deliberate design or redesign of their supply chains with sustainability in mind (Carter et al. 2019). Especially intermediary SMEs have shown to be critical players in developing supply-chain resilience (Weber and Wiek 2021).

In summary, there are strong symbiotic relationships between the resilience of SMEs and their communities, and actions that strengthen SMEs often directly translate into amplifying resilience in the wider community.

## From firm-focused sustainability policy to enabling transformations in private sector activity systems

Considering the pivotal role that SMEs can play in building community resilience, it is important to explore specific ways to enhance SMEs’ resilience and leverage their amplification potential. A promising set of instruments include targeted policies issued through local, regional, or federal governments. Governments have developed numerous policies to support SMEs such as economic incentives, tax relief, subsidies to address SMEs’ resource constraints; or through policies that facilitate resilience-specific training and development programs to address SMEs’ knowledge and skill gaps; or through policies that reduce the time-to-market of resilience innovations to mitigate SMEs’ limited innovation leverage (Amankwah-Amoah and Syllias 2020; Halkos et al. 2018; Price et al. 2013). The effectiveness of these policies will depend heavily on the size and sector of the SME.

Under the broader perspective of resilience and sustainability, additional policies become relevant, namely those that support SMEs in adopting sustainable business practices and models (Burch et al. 2020). Such policies may take a relational view of SME, as part of a more complex and wide-ranging private sector activity system (Burch and Di Bella 2021). This includes policies that provide sustainable seed capital or loan guarantees, facilitate sustainable innovation, or provide favorable regulation to sustainable SMEs, including regulations for zoning, public procurement (incentive contracts), local marketing, or waste management (Sharma et al. 2021; Walker and Preuss 2008). Prohibitive policies might also show effects, but only if consistently enforced (Wilson et al. 2012).

Government policy intervention alone, through financial incentives or otherwise, will not anchor resilience in SMEs and the communities they are embedded in. While government policies offer valuable support, multi-stakeholder support is emerging, too, in which government plays a leading or collaborating role together with banks (e.g., social finance), universities (e.g., incubators or labs), or consultancies (e.g., training programs) (cf. Hansen and Klewitz 2017).

Recent research suggests that there are untapped opportunities for involving SMEs more directly in policy-making and other governance efforts to advance community resilience (Westman et al. 2021). Innovation policy studies suggests that a wide range of stakeholder groups, including SMEs and government agencies, need to cooperate in a number of transformational activities to achieve this goal, including jointly setting long-term goals; seeing opportunity in challenges and crises; mobilizing businesses to engage in transformative innovation; and improving policy coordination (Fagerberg 2018). This may be more likely among larger, medium-sized firms, however which have increased capacity to engage in lobbying and consultation efforts.

## The emergence of a new research agenda for accelerating sustainability transformations and community resilience through small firms

The complex web of macro-economic trends, hardened infrastructure, short-sighted corporate practices, and ingrained consumption habits, which has led inexorably to unsustainable outcomes worldwide, has been the subject of intense analysis over the last two decades. However, being mostly problem-oriented, this body of literature offers little guidance (with some important exceptions including Gleidt et al., 2018; Loorbach and Wijsman 2013; Schaltegger et al. 2020) on the specific approaches that should be taken to strengthen and amplify sustainability transformations among vulnerable (but innovative) sectors of the economy.

The insights into the drivers of SMEs’ resilience, and the effects on broader community resilience, illustrate such research efforts; yet, considering various knowledge gaps, they also highlight the need for a new and much more engaged research agenda. Such an agenda could support ongoing recovery efforts as the COVID-19 pandemic continues to unfold and the longer-term fallout becomes apparent.

Understanding what is required to build resilience in and through SMEs calls for a new type of transdisciplinary action research agenda. Such an agenda would center on SMEs as pivotal actors and amplifiers for community resilience and would link (1) case studies on resilient SMEs and their pathway to resilience (cf. Burch et al. 2020) to (2) collaborative capacity-building activities in SMEs (in collaboration with government agencies and consultancies) and to (3) experimentation in SMEs that allows for fail-safe adoption of resilient business practices and models. In parallel, research would need to (4) explore and comparatively test government policies and public-private partnerships in support of SMEs resilience and, by extension, the resilience of the wider community.

Transdisciplinary scholars will need to engage in more meaningful and sustained ways with SMEs to better understand the steps, sequences, and limits in their pursuit of sustainability and resilience-building practices.

## Data Availability

As this is a Perspective article that is conceptual rather than empirical in nature, there are no accompanying data nor materials.

## Footnotes

1 We define an SME as a firm with fewer than 500 employees, in any sector.

## References

[CR1] Ahn JM, Mortara L, Minshall T (2018). Dynamic capabilities and economic crises: has openness enhanced a firm's performance in an economic downturn?. Ind Corp Chang.

[CR2] Alcalde-Heras H, Iturrioz-Landart C, Aragon-Amonarriz C (2019). SME ambidexterity during economic recessions: the role of managerial external capabilities. Manag Decis.

[CR3] Aldrich DP, Meyer MA (2015). Social capital and community resilience. Am Behav Sci.

[CR4] Amankwah-Amoah J, Syllias J (2020). Can adopting ambitious environmental sustainability initiatives lead to business failures? An analytical framework. Bus Strateg Environ.

[CR5] Andres L, Round J (2015). The creative economy in a context of transition: a review of the mechanisms of micro-resilience. Cities.

[CR6] Bakos J, Siu M, Orengo A, Kasiri N (2020). An analysis of environmental sustainability in small & medium-sized enterprises: patterns and trends. Bus Strateg Environ.

[CR7] Balasubramanian S, Shukla V, Mangla S, Chanchaichujit J (2021). Do firm characteristics affect environmental sustainability? A literature review-based assessment. Bus Strateg Environ.

[CR8] Belitski M, Guenther C, Kritikos AS, Thurik R (2022). Economic effects of the COVID-19 pandemic on entrepreneurship and small businesses. Small Bus Econ.

[CR9] Birchall J, Ketilson LH (2009). Resilience of the cooperative business model in times of crisis.

[CR10] Burch S, Di Bella J (2021). Business models for the Anthropocene: accelerating sustainability transformations in the private sector. Sustain Sci.

[CR11] Burch S, DiBella J, Rao-Williams J, Forrest N, Hermelingmeier V, Ninomiya SDM, Chisholm K (2020). Sustainable business practices can build resilient local economies for a post-COVID-19 recovery. TRANSFORM project paper No. 1.

[CR12] Caldera HTS, Desha C, Dawes L (2019). Evaluating the enablers and barriers for successful implementation of sustainable business practice in ‘lean’SMEs. J Clean Prod.

[CR13] Carter CR, Hatton MR, Wu C, Chen X (2019). Sustainable supply chain management: continuing evolution and future directions. Int J Phys Distribut Logist Manage.

[CR14] DiBella J, Forrest N, Burch S, Rao‐Williams J, Ninomiya SM, Hermelingmeier V, Chisholm K. Exploring the potential of SMEs to build individual, organizational, and community resilience through sustainability‐oriented business practices. Bus Strategy Environ. 2022.

[CR15] Elmqvist T, Andersson E, Frantzeskaki N, McPhearson T, Olsson P, Gaffney O, Takeuchi K, Folke C (2019). Sustainability and resilience for transformation in the urban century. Nat Sustain.

[CR16] Fagerberg J (2018). Mobilizing innovation for sustainability transitions: a comment on transformative innovation policy. Res Policy.

[CR17] Fairlie, R. W. (2020). The impact of Covid-19 on small business owners: evidence of early-stage losses from the April 2020 current population survey (No. w27309). National Bureau of Economic Research.

[CR18] Folke C (2006). Resilience: the emergence of a perspective for social-ecological systems analysis. Global Environ Change.

[CR19] Fraser T, Page-Tan C, Aldrich DP. Won’t you be my neighbor? Uncovering ties between social capital and COVID-19 outcomes at local levels. SSRN Electron J. 2021. 10.2139/SSRN.3788540.

[CR20] Gliedt T, Hoicka CE, Jackson N (2018). Innovation intermediaries accelerating environmental sustainability transitions. J Clean Prod.

[CR21] Halkos G, Skouloudis A (2020). Investigating resilience barriers of small and medium-sized enterprises to flash floods: a quantile regression of determining factors. Clim Dev.

[CR22] Halkos G, Skouloudis A, Malesios C, Evangelinos K (2018). Bouncing back from extreme weather events: some preliminary findings on resilience barriers facing small and medium-sized enterprises. Bus Strateg Environ.

[CR23] Klewitz J, Hansen EG, Wagner S (2017). Publicly mediated inter-organisational networks: a solution for sustainability-oriented innovation in SMEs?. Entrepreneurship, innovation and sustainability.

[CR24] Holling CS (1973). Resilience and stability of ecological systems. Annu Rev Ecol Syst.

[CR25] Howard M, Böhm S, Eatherley D (2022). Systems resilience and SME multilevel challenges: a place-based conceptualization of the circular economy. J Bus Res.

[CR26] Linnenluecke MK (2017). Resilience in business and management research: a review of influential publications and a research agenda. Int J Manag Rev.

[CR27] Loorbach D, Wijsman K (2013). Business transition management: exploring a new role for business in sustainability transitions. J Clean Prod.

[CR28] Lu Y, Wu J, Peng J, Lu L (2020). The perceived impact of the Covid-19 epidemic: evidence from a sample of 4807 SMEs in Sichuan Province, China. Environmental Hazards.

[CR29] McNaughton RB, Gray B (2017). Entrepreneurship and resilient communities. J Enterprising Commun.

[CR30] Price L, Rae D, Cini V (2013). SME perceptions of and responses to the recession. J Small Bus Enterp Dev.

[CR31] Robinson J, Kengatharan N (2020). Exploring the effect of Covid-19 on small and medium enterprises: early evidence from Sri Lanka. J Appl Econ Business Res.

[CR32] Ryu J, Yoon EJ, Park C, Lee DK, Jeon SW (2017). A flood risk assessment model for companies and criteria for governmental decision-making to minimize hazards. Sustainability.

[CR33] Schaltegger S, Hörisch J, Loorbach D (2020). Corporate and entrepreneurial contributions to sustainability transitions. Bus Strateg Environ.

[CR34] Scoones I, Stirling A, Abrol D, Atela J, Charli-Joseph L, Eakin H, Yang L (2020). Transformations to sustainability: combining structural, systemic and enabling approaches. Curr Opin Environ Sustain.

[CR35] Sharma NK, Govindan K, Lai KK, Chen WK, Kumar V (2021). The transition from linear economy to circular economy for sustainability among SMEs: a study on prospects, impediments, and prerequisites. Bus Strateg Environ.

[CR36] Steiner A, Atterton J (2014). The contribution of rural businesses to community resilience. Local Econ.

[CR37] Stirling A (2015). Emancipating transformations: from controlling ‘the transition’ to culturing plural radical progress.

[CR38] Thomas A, Francis M, John E, Davies A (2012). Identifying the characteristics for achieving sustainable manufacturing companies. J Manuf Technol Manag.

[CR39] Walker H, Preuss L (2008). Fostering sustainability through sourcing from small businesses: public sector perspectives. J Clean Prod.

[CR40] Weber H, Wiek A (2021). Cooperating with ‘open cards’ – the role of small intermediary businesses in realizing sustainable international coffee supply. Frontiers in in Sustainable Food Systems.

[CR41] Westley F, Olsson P, Folke C, Homer-Dixon T, Vredenburg H, Loorbach D, Thompson J, Nilsson M, Lambin E, Sendzimir J, Banerjee B, Galaz V, Leeuw S (2011). Tipping toward sustainability: emerging pathways of transformation. AMBIO.

[CR42] Westman L, Moores E, Burch S. Bridging the governance divide: understanding the role of SMEs in urban sustainability interventions. Cities. 2021;108:102944.

[CR43] Wilson CD, Williams ID, Kemp S (2012). An evaluation of the impact and effectiveness of environmental legislation in small and medium-sized enterprises: experiences from the UK. Bus Strateg Environ.

